# How metaverse-enabled digital transformation drives sustainable supply chain innovation: evidence from Pakistan’s textile industry

**DOI:** 10.1038/s41598-026-40819-6

**Published:** 2026-03-11

**Authors:** Muhammad Abdullah, Aman Ullah, Muhammad Farooq Jamal, Eman Abdullah Aldakheel, Doaa Sami Khafaga, Sana Ashraf, Zainab Rasool

**Affiliations:** 1 Faisalabad Business School, National Textile University, Faisalabad, Pakistan; 2 Research Institute of Business Analytics and Supply Chain Management, College of Management, Shenzhen University, Shenzhen, 518060 China; 3 Department of Computer Sciences, College of Computer and Information Sciences, Princess Nourah bint Abdulrahman University, P.O. Box 84428, Riyadh, 11671 Saudi Arabia; 4 College of Computer Science and Software Engineering, Shenzhen University, Shenzhen, 518060 China; 5 School of Arts & Design, National Textile University, Faisalabad, Pakistan

**Keywords:** Metaverse capabilities, Green process reconfiguration, Eco-intelligent decision support, Digital maturity, Sustainable supply chain innovation, Pakistan textile industry, Business and management, Business and management, Information systems and information technology, Science, technology and society

## Abstract

Despite the growing urgency of sustainability, many firms continue to rely on static systems and incremental improvements that fall short of enabling deep transformation. Drawing on the dynamic capabilities theory (DCT) and the technology–organization–environment (TOE) framework, this research proposes a conceptual model in which the metaverse-enabled digital twin integration (DTI) and virtual supply chain visibility (VSCV) influence sustainable supply chain innovation (SSCI) through the mediating roles of green process reconfiguration (GPR) and eco-intelligent decision support (EIDS), with digital maturity (DM) as a moderating factor. Data were collected from 430 supply chain professionals in the Pakistan’s textile industry using a structured survey and analyzed using structural equation modeling with WarpPLS. Results show that DTI and VSCV significantly contribute to SSCI via GPR and EIDS, and that the impact of these internal mechanisms is amplified by higher levels of DM. Firm size and export orientation were included as control variables to account for structural and contextual differences across firms. This research enriches the literature by identifying how immersive and interactive digital tools, when embedded within adaptive organizational capabilities, can drive meaningful sustainability transformation. It offers practical insights for firms and policymakers seeking to align digitalization with environmental goals.

**Supplementary Information:**

The online version contains supplementary material available at 10.1038/s41598-026-40819-6.

## Introduction

In recent years, the concept of sustainable supply chain management (SSCM) has emerged as a critical strategic priority for firms seeking to align operational efficiency with environmental and social responsibility^1,2^. As global climate concerns intensify, organizations across industries are increasingly pressured by regulators, consumers, and investors to adopt greener practices throughout their supply chains from sourcing and production to distribution and end-of-life product handling^2^. The integration of sustainability into supply chain operations is no longer a peripheral concern; it is now central to achieving long-term competitiveness, resilience, and compliance with emerging environmental standards such as carbon neutrality goals and circular economy principles^3^. Despite the growing focus on SSCM, many organizations, especially in resource-intensive and operationally complex sectors continue to face significant challenges. Key among these are the inability to redesign existing supply chain processes to reduce environmental impact^4^, and the lack of timely^3^, data-driven decision-making capabilities that incorporate sustainability metrics^1^. Traditional systems often fall short in enabling firms to experiment with alternative processes, simulate sustainability trade-offs, or access real-time environmental data across multiple supply chain tiers^5–9^. These gaps persist even in firms that have adopted baseline digital technologies, suggesting that more advanced, integrated, and dynamic solutions are required to support sustainable transformation at both the process and decision levels.

Over the past decade, a growing body of research has explored the role of digital technologies in enhancing the sustainability of supply chain operations. Technologies such as enterprise resource planning (ERP) systems^9^, internet of things (IoT)^7^, big data analytics (BDA)^8^, and blockchain have been extensively studied for their potential to improve transparency, traceability, and operational efficiency^5,6^. For example, ERP and BDA have been shown to support sustainability reporting and performance monitoring^10,11^, while IoT and blockchain technologies have enabled real-time tracking of emissions, inventory flows, and supplier compliance^6^. Several scholars have emphasized that these technologies contribute meaningfully to reducing waste, improving resource allocation, and strengthening sustainability governance mechanisms across global supply chains^11–14^. However, despite these contributions, scholars have debated the extent to which such technologies can generate transformative rather than incremental improvements in sustainability outcomes. Many existing studies highlight that while digital tools enhance data visibility, they often lack the integrative and predictive capabilities required for real-time experimentation and strategic environmental decision-making. While these technologies offer important foundational capabilities, they remain limited in their ability to simulate, reconfigure, or test sustainable supply chain scenarios dynamically. Most systems are designed for linear data processing, lacking the flexibility to model complex trade-offs or anticipate the impact of alternative green strategies before implementation^5^. Moreover, traditional systems often fail to support decision-making environments where operational, financial, and environmental data are integrated in real time for sustainability focused planning^7^. These limitations highlight a technological gap in current digital transformation efforts: existing solutions are often static, fragmented, or reactive failing to enable proactive, interactive, and innovation-driven sustainability interventions at both the strategic and operational levels. Addressing this gap requires more immersive, real-time, and process-integrated.

To address these persistent limitations, this study proposes the integration of two core metaverse-driven capabilities, digital twin integration (DTI) and virtual supply chain visibility (VSCV) as advanced enablers of sustainable supply chain innovation (SSCI) through the lens of the technology–organization–environment (TOE) framework^15^ and dynamic capabilities theory (DCT)^16^. Within the TOE framework, DTI and VSCV represent the technological dimension by enabling real-time simulation and immersive visibility of supply chain processes. The organizational dimension is reflected through internal transformation mechanisms, namely GPR and EIDS, which capture firms’ abilities to redesign processes and integrate environmental intelligence into decision-making. The environmental dimension is represented by sustainability-related pressures, including regulatory requirements, buyer-driven environmental expectations, and competitive dynamics in global supply chains. Within this study, DTI represents the technological dimension by creating real-time digital replicas of physical supply chain processes, enabling the simulation of alternative production setups, logistics flows, and resource configurations without disrupting actual operations^17^. This capability empowers decision-makers to virtually test the environmental, operational, and financial implications of green strategies before deployment, significantly reducing the risk and cost of experimentation^17^. Meanwhile, VSCV offers a high-fidelity, interactive view of the entire supply chain network, including upstream and downstream partners, allowing for enhanced traceability, transparency, and coordination^18^. By integrating real-time data streams into immersive visual environments, this visibility promotes faster and more informed responses to sustainability risks and opportunities^18^. Together, these capabilities move beyond the static and siloed nature of traditional systems, offering a proactive and immersive approach to sustainability. They help firms anticipate disruptions, assess sustainability trade-offs, and align operations with environmental goals, laying the groundwork for innovation-driven supply chain improvement.

One of the key ways advanced digital capabilities contribute to sustainability is by enabling firms to simulate and optimize existing supply chain processes in real time^19^. Through digital modeling, managers can replicate production flows, energy use, and logistics to identify inefficiencies and test eco-friendly alternatives before implementation^18^. This fosters proactive redesigns that are less resource-intensive and more circular^20^. This transformation, termed green process reconfiguration (GPR), reflects a firm’s ability to redesign operations for improved environmental performance. It arises from the intersection of digital transformation and sustainability, showing how advanced digital capabilities drive eco-focused process redesign. Such reconfiguration lays the foundation for SSCI by embedding environmental efficiency into core processes while enhancing decision-making through real-time data, sustainability metrics, and predictive insights. Managers can evaluate environmental impacts alongside cost and risk in their decisions, shifting from reactive reporting to strategic sustainability planning^20^. This is captured as eco-intelligent decision support (EIDS), which uses integrated, sustainability-focused analytics to guide operations. EIDS arises from the convergence of business analytics and environmental sustainability, highlighting how data intelligence supports environmentally responsible decisions. This empowers firms to innovate by embedding ecological intelligence directly into supply chain decisions, advancing sustainability-driven innovation.

While advanced digital capabilities can enable process redesign and intelligent decision-making, their effectiveness often depends on the broader technological and organizational readiness of the firm^21^. Not all organizations possess the internal capacity to fully leverage such technologies; in many cases, the lack of integrated systems, digital skills, or a supportive innovation culture limits the actual impact of even the most powerful tools^22^. In this context, firms that are more digitally mature are better equipped to absorb, adapt, and apply emerging technologies in ways that drive meaningful change^23^. They are more likely to have the infrastructure, leadership support, and cross-functional alignment required to operationalize digitally enabled sustainability strategies^24^. This study captures this conditional influence through DM, defined as a firm’s capability to deploy, integrate, and scale digital technologies across supply chain functions. DM encompasses technical readiness, strategic alignment, and cultural adaptability, which together sustain digital transformation efforts^23^. In more digitally mature firms, the benefits of GPR and EIDS are expected to be more fully realized, thus strengthening their contribution to SSCI. The theoretical grounding for this moderating role is drawn from the DCT, which posits that organizations must continually reconfigure internal competencies and resources to respond effectively to changing environments^16,25^. Grounded in the DCT and TOE frameworks, this study proposes the following research questions.

*RQ1: How do metaverse capabilities*,* specifically digital twin integration and virtual supply chain visibility*,* influence sustainable supply chain innovation?*

*RQ2: To what extent do green process reconfiguration and eco-intelligent decision support mediate the relationship between metaverse capabilities and sustainable supply chain innovation?*

*RQ3: How does digital maturity moderate the relationship between the mediating variables (green process reconfiguration and eco-intelligent decision support) and sustainable supply chain innovation?*

This study is situated within the context of Pakistan’s textile industry, a sector that forms the backbone of the national economy in terms of employment, exports, and industrial output^26^. Despite its significance, the industry faces persistent challenges in aligning its supply chain operations with sustainability goals^27^. Many textile firms operate with low visibility across supply chain tiers, rely on outdated and resource-intensive processes, and lack real-time data integration to support environmentally informed decision-making^28^. While some have adopted basic digital tools, the absence of advanced capabilities such as simulation, dynamic process redesign, and integrated decision support severely limits their ability to innovate sustainably^29^. Additionally, variation in DM across firms creates a disparity in how effectively digital interventions translate into tangible sustainability outcomes^30^. These structural and digital constraints also make the textile industry a theoretically appropriate setting for examining advanced digital technologies, as firms with limited legacy system integration may adopt immersive and simulation-based solutions through technological leapfrogging logic, bypassing incremental digitalization stages. These issues align directly with the gaps identified earlier and motivate the research questions, which examine how advanced digital capabilities, internal process transformations, and organizational readiness collectively enable sustainable supply chain innovation in the Pakistani textile context.

By addressing the proposed research questions, this study makes several key contributions. First, it extends the digital transformation and sustainability literature by introducing metaverse capabilities, specifically DTI and VSCV as critical technological enablers of SSCI. Second, this research advances theory by identifying GPR and EIDS as novel mechanisms that explain how digital capabilities translate into environmental innovation. Third, by integrating DM as a moderating variable, the study adds a dynamic, firm-level lens to understand when and where digital transformation yields the greatest sustainability impact. This theoretical contribution is grounded in both the DCT and the TOE framework, the latter explains how technological, organizational, and environmental contexts jointly influence technology adoption and sustainable outcomes. Finally, the study contributes empirically by focusing on Pakistan’s textile industry, offering new insights from an emerging economy context where sustainable digital transformation remains underexplored yet critically needed.

The paper is structured as follows. "Literature review and theoretical insights" reviews the literature on SSCI and digital transformation and develops the conceptual framework and hypotheses. "Hypothesis formulation" outlines the theoretical foundation, while "Research methodology" details the methodology. "Data evaluation and findings" presents empirical results, "Discussion" discusses key findings, and "Conclusion" concludes with implications and future research directions.

## Literature review and theoretical insights

### Sustainable supply chain innovation

Sustainable supply chain has emerged as a critical area of inquiry as firms seek to balance environmental responsibility with operational excellence^2^. Existing literature emphasizes that integrating sustainability into supply chain practices can enhance resource efficiency, reduce ecological impact, and improve long-term competitiveness^1,28^. Scholars have highlighted various enablers of sustainable supply chain, including internal leadership commitment, external stakeholder collaboration, regulatory compliance, and knowledge sharing across the supply chain network^6,13,14^. Additionally, innovation within sustainable supply chains has been linked to improved resilience, cost savings, and reputation management, making it a strategic priority for firms in both developed and emerging economies^7,18^.

However, despite these advances, current research faces limitations in explaining how firms can move from sustainability intent to sustainability execution in a systematic and innovation-driven manner. Much of the literature focuses on reporting, compliance, and incremental improvements, while overlooking how firms can experiment with alternative designs, simulate sustainability-driven changes, or visualize interconnected impacts across the supply chain^31,32^. Moreover, existing models often lack the ability to support real-time environmental decision-making or offer dynamic views of supply chain performance that enable proactive responses to sustainability challenges^33,34^. Recent studies emphasize that sustainability-oriented innovation depends not only on internal process improvement but also on integrating external knowledge across supply chain tiers. Reference^35^ show that global knowledge integration enhances green product innovation through environmental product design and cross-functional collaboration. However, while such research highlights inter-firm collaboration, it overlooks how digital and immersive technologies can internalize these insights. This study addresses that gap by examining how metaverse-enabled capabilities translate sustainability intent into actionable innovation.

### Digital twin integration

Past research has extensively examined the role of digital technologies such as ERP systems^9^, the IoT^6^, big data analytics, and blockchain in supporting SSCI^5,6^. These technologies have contributed to improvements in transparency, data availability, and process automation across supply chain networks. For example, ERP systems have enhanced internal coordination for environmental compliance, while IoT and blockchain have facilitated real-time monitoring of emissions, energy usage, and product traceability^5–7^. Big data analytics, in turn, has enabled firms to track and evaluate sustainability metrics for improved decision-making. Despite these benefits, most of these technologies are limited in their ability to dynamically model and test supply chain scenarios in advance^8^. They often lack the immersive, real-time simulation capabilities needed to experiment with environmentally sustainable alternatives or predict how changes in one part of the supply chain might impact others. Additionally, they provide limited support for virtual experimentation, cross-process synchronization, or system-wide redesigns aligned with sustainability goals.

To address these limitations, recent advancements have focused on DTI as a core metaverse capability, a technology that creates real-time digital replicas of physical assets, systems, and processes within the supply chain^17^. DTI allows firms to simulate various configurations of production, logistics, and resource allocation, enabling managers to test the environmental, financial, and operational consequences of proposed changes before actual implementation^36^. This virtual experimentation environment supports the identification of optimal sustainability strategies, reduces the risks associated with green innovations, and allows for rapid prototyping of supply chain reconfigurations^37^. By offering a dynamic and visual model of the end-to-end supply chain, DTI helps organizations anticipate disruptions, optimize material flows, and integrate environmental considerations into core decision-making processes^38^. Ultimately, this capability plays a foundational role in driving SSCI, as it empowers firms to proactively design and validate more sustainable operations with greater precision and confidence. Recent evidence highlights the transformative role of digital technologies in enhancing supply chain resilience. Reference^39^ show that blockchain-based systems improve transparency and risk management, strengthening firms’ recoverability. Building on this, the present study examines how digital twin integration drives proactive, simulation-based sustainability innovation beyond reactive recovery.

### Virtual supply chain visibility

Supply chain visibility has been widely acknowledged as a foundational element in enhancing both performance and sustainability across supply chains. Existing research has demonstrated that digital technologies such as IoT-enabled devices, RFID systems, and cloud-based monitoring tools have contributed to greater visibility by allowing real-time tracking of inventory levels, shipment status, emissions, and supplier activities^40^. These advancements have supported more informed sourcing decisions, improved logistics coordination, and enhanced compliance with environmental regulations^8^. However, many of these visibility solutions remain limited in scope^7^. They tend to deliver fragmented or delayed data, focus on individual supply chain nodes, and lack a unified system-wide representation of sustainability performance^7^. As a result, decision-makers often face challenges in obtaining timely, actionable insights across the entire supply chain network^7^. In addition, most existing systems are reactive in nature, providing alerts or insights only after sustainability risks or performance deviations have occurred, rather than enabling proactive and strategic interventions^5^.

To address these limitations, this study introduces VSCV as a core metaverse capability that extends traditional visibility frameworks through immersive, interactive, and system-wide data integration^18,41^. VSCV leverages real-time data streams, advanced analytics, and spatial visualizations to offer an integrated view of end-to-end supply chain operations^42^. Unlike conventional tools, this capability enables managers to trace environmental performance indicators, track sustainability compliance across multiple tiers, and coordinate responses to disruptions in real time^8^. The virtual and visual nature of this capability promotes better communication among stakeholders and supports predictive planning by illustrating the downstream and upstream effects of key decisions^43^. By offering a synchronized and real-time visualization of supply chain activities, VSCV empowers firms to respond more effectively to environmental risks, improve traceability, and embed sustainability considerations into operational planning^44^. In doing so, it plays a vital role in advancing SSCI.

### Green process reconfiguration

While sustainable supply chain increasingly serves as a strategic imperative, most supply chain operations remain grounded in traditional process structures that are rigid, resource-intensive, and slow to adapt to sustainability demands^31,32^. Existing research in sustainable supply chain management has largely focused on initiatives such as compliance enforcement, emission control, and supplier monitoring to reduce environmental harm^1,2,6^. While these measures have yielded measurable results, they often rely on incremental improvements within prestructured workflows, offering little opportunity for experimentation or structural adaptation^33,34^. Consequently, many firms lack the operational flexibility to respond dynamically to environmental disruptions or to proactively redesign their processes to align with evolving sustainability standards. Although digital technologies such as DTI and VSCV provide simulation and real-time modeling capabilities, their effectiveness in supporting sustainability-driven innovation depends heavily on a firm’s internal ability to act on the insights they generate^42,45^. Without the capacity to modify production configurations, logistics setups, or sourcing strategies in line with ecological goals, the transformative potential of these technologies remains underutilized^46^. This signals a critical gap in literature: a missing process-based capability that connects digital innovation to sustainability-oriented operational change.

To address these limitations, under the lens of DCT^16^, this study conceptualizes GPR as a distinct, digitally enabled process capability. Drawing on DCT’s emphasis on sensing environmental challenges and reconfiguring internal resources^25^, GPR captures a firm’s ability to strategically redesign core supply chain processes, such as production, logistics, and sourcing, to enhance environmental performance while maintaining or improving operational efficiency. It emphasizes structural flexibility, environmental responsiveness, and proactive experimentation. Unlike conventional process optimization, GPR enables firms to simulate, test, and implement alternative, more sustainable process configurations using advanced digital insights. Serving as a mediating mechanism, GPR explains how metaverse-enabled capabilities are transformed into sustainability-oriented innovation through the reconfiguration of core operational processes.

### Eco-intelligent decision support

A critical enabler of sustainable supply chain lies not only in reconfiguring physical processes but also in enhancing the quality of decision-making across supply chain functions^47^. Traditional decision-making frameworks in supply chains often rely on fragmented data, reactive reporting, and narrow performance metrics that prioritize cost and speed over environmental outcomes^31,32^. Although the adoption of technologies such as ERP systems, big data analytics, and IoT has improved operational visibility and efficiency, these tools frequently fall short in providing integrated, sustainability-oriented intelligence for strategic planning and execution^9^. As a result, firms often struggle to incorporate environmental considerations into real-time decisions regarding sourcing, production planning, logistics, and risk mitigation^33,34^. The absence of advanced decision support mechanisms that can integrate environmental metrics with operational and financial data remains a significant barrier to achieving systemic innovation in supply chain sustainability^7,8^. Recent advancements in immersive technologies such as DTI and VSCV offer the potential to overcome these limitations by enabling real-time simulation, multi-dimensional visualization, and predictive modeling^41,42,45^. However, these technologies alone do not guarantee improved decision quality unless firms develop the cognitive and analytical capacity to interpret and act upon the environmental intelligence these systems provide^24^. This underscores the need for a mechanism that connects digital insight with environmentally informed decision-making.

To address these limitations, under the lens of DCT^16^, this study conceptualizes EIDS as a distinct, digitally enabled decision-making capability. Drawing on DCT’s emphasis on sensing environmental signals and reconfiguring cognitive and analytical resources^25^, EIDS captures a firm’s ability to integrate real-time environmental, operational, and predictive data into supply chain decision processes to support sustainability-oriented action. It reflects a proactive, analytics-driven orientation in which environmental intelligence is embedded into strategic and operational decisions rather than treated as an ex-post evaluation. Unlike conventional decision support systems, EIDS enables managers to interpret digital insights holistically and coordinate sustainability-aligned decisions across supply chain functions. Serving as a mediating mechanism, EIDS explains how metaverse-enabled capabilities are translated into sustainable supply chain innovation by aligning decision architectures with ecological objectives and long-term competitiveness.

### Digital maturity

The success of digital transformation in enabling sustainable supply chain is not solely dependent on the adoption of advanced technologies^24^. It also hinges on an organization’s ability to absorb, integrate, and apply these technologies effectively across its processes and decision-making structures^48^. Prior studies have shown that even when powerful digital tools such as simulation platforms, analytics engines, or visibility dashboards are implemented, their actual impact on sustainability outcomes varies widely across firms^21,22^. In many cases, firms encounter internal barriers such as fragmented data infrastructures, limited digital literacy, and cultural resistance to change that inhibit the full realization of digital benefits^22^. These disparities point to the critical role of organizational readiness and capability in determining how technology is leveraged for sustainability.

In this context, DM emerges as a key organizational characteristic that shapes the extent to which digital innovations translate into strategic outcomes^23^. Firms with high digital maturity tend to exhibit stronger alignment between their technology infrastructure, business strategy, and innovation culture. They are more capable of deploying technologies on a scale, integrating data across functions, and supporting cross-functional collaboration in sustainability-focused projects^22^. These firms are also more likely to respond proactively to environmental challenges by embedding ecological considerations into their digital roadmaps^24^. Therefore, DM is expected to influence the strength and effectiveness of the relationship between internal transformation mechanisms, such as GPR and EIDS and the achievement of SSCI.

### Theoretical foundation

This study is grounded in two complementary theoretical lenses: DCT and the TOE. These theories provide a robust foundation for understanding how digital capabilities interact with internal organizational processes and contextual readiness to drive SSCI.

#### Dynamic capabilities theory

DCT emphasizes an organization’s capacity to purposefully create, extend, or modify its resource base in response to rapidly changing environments^16^. In the context of SSCI, firms must continuously reconfigure their operations and decision-making structures to address evolving environmental demands, regulatory pressures, and stakeholder expectations^49^. DCT highlights that competitive advantage is not solely derived from static resources but from the ability to adapt and transform those resources into new configurations aligned with strategic goals^16,25^.

In this study, DCT supports the inclusion of two mediating constructs GPR and EIDS which represent dynamic capabilities through which digital technologies influence sustainability outcomes. GPR reflects a firm’s ability to redesign core operations to align with ecological objectives, while EIDS captures the integration of environmental intelligence into strategic and operational decisions. Both constructs embody the DCT emphasis on adaptability, learning, and continuous transformation as essential to sustainability-driven innovation. DM, positioned as a moderating variable, is also grounded in DCT^49^. It reflects a firm’s readiness and capacity to integrate, scale, and align digital technologies with broader innovation and sustainability strategies. From a DCT perspective, DM enhances a firm’s absorptive and transformative capabilities, enabling it to unlock greater value from digital investments and respond more effectively to complex environmental challenges.

#### Technology–organization–environment framework

TOE provides a multi-dimensional view of the factors that influence technology adoption and implementation^15^. TOE highlights that technological innovation is shaped by three key contexts: (1) the technological characteristics of the innovation, (2) organizational readiness, and (3) external environmental pressures. This framework is particularly relevant to SSCI, where technological capabilities must be supported by organizational structures and contextual conditions.

In this study, the technological context is represented by the metaverse-driven capabilities DTI and VSCV, which provide simulation, modeling, and visibility functionalities necessary for sustainable transformation. The organizational context is reflected through internal mechanisms such as GPR and EIDS, as well as through DM as an indicator of internal readiness. The environmental context is embedded in the sustainability pressures faced by firms, particularly in resource-intensive industries such as textiles.

Together, DCT and TOE offer a holistic theoretical foundation for examining how digital transformation efforts when aligned with internal capabilities and contextual readiness can generate SSCI. DCT explains the need for internal transformation mechanisms, while TOE frames the broader conditions under which technology adoption leads to impactful outcomes.

## Hypothesis formulation

### Digital twin integration and green process reconfiguration

To achieve sustainability-oriented innovation in supply chains, firms must move beyond surface-level efficiency enhancements and develop capabilities that enable structural and process-level transformation^49^. Traditional supply chain processes often lack the flexibility to accommodate evolving environmental goals, as they are designed for linear execution, cost-efficiency, and stability^31,32^. These rigid systems are poorly suited for experimentation, testing of green alternatives, or dynamic redesign of production and logistics workflows^49^. This gap in the literature highlights the need for a new construct that captures a firm’s capacity to actively reshape its operational architecture in response to environmental objectives, what this study conceptualizes as GPR.

DTI, a core metaverse capability, offers the technological foundation to support such transformation^36^. By creating real-time, virtual replicas of physical supply chain systems, DTI enables firms to simulate alternative configurations, model environmental impacts, and test operational scenarios without disrupting actual workflows^45^. This capability empowers decision-makers to assess sustainability trade-offs, anticipate risks, and redesign processes based on predictive insights^50^. Through this iterative and data-informed experimentation, firms are more likely to engage in meaningful reengineering of their processes aligned with sustainability goals^44^. Hence, DTI is expected to enhance GPR as a dynamic capability that drives sustainability-oriented innovation. This relationship aligns with DCT, which posits that firms achieve sustained innovation by continuously reconfiguring their operational processes in response to environmental and technological changes^49^.

H1a: Digital twin integration positively influences green process reconfiguration.

### Digital twin integration and eco-intelligent decision support

As supply chains increasingly aim to align operational decisions with environmental priorities, firms must develop the capacity to integrate ecological metrics into their planning and control systems^51^. However, many traditional decision-making frameworks in supply chains are constrained by reactive approaches, fragmented data systems, and narrow performance indicators that focus primarily on cost, time, or quality^7,8^. These limitations prevent firms from systematically embedding environmental considerations into their decisions^5^. To address this gap, there is a growing need for a construct that captures an organization’s ability to make data-informed, sustainability-driven choices across operational levels, this study introduces this as EIDS.

DTI, by enabling real-time replication of supply chain processes, creates a rich digital environment where diverse data streams including environmental, operational, and financial inputs, which can be fused for advanced analytics and predictive modeling^36^. Through dynamic visualization and what-if scenario analysis, DTI empowers managers to evaluate the environmental impact of decisions before implementation^38^. This capability shifts decision-making from reactive to proactive, embedding sustainability logic into planning, procurement, and execution^36^. As a result, firms become more capable of integrating ecological intelligence into their decision infrastructure, thereby cultivating EIDS. This relationship is consistent with DCT, which emphasizes that firms achieve adaptive advantage by continuously reconfiguring decision-making routines to align with environmental and technological shifts^49^.

H1b: Digital twin integration positively influences EIDS.

### Virtual supply chain visibility and green process reconfiguration

The complexity of modern supply chains, combined with growing environmental pressures, requires firms to continuously adapt and reconfigure their operational structures^51^. However, traditional supply chains often operate with limited visibility across upstream and downstream activities, which constrains a firm’s ability to identify inefficiencies, environmental bottlenecks, or opportunities for sustainable redesign^49^. Without clear visibility into the extended supply network, firms face significant barriers when attempting to implement process changes that align with sustainability objectives^31^. This further reinforces the need for GPR, a capability that captures a firm’s ability to redesign operational workflows in response to environmental goals.

VSCV, a metaverse-enabled capability, provides a high-fidelity, interactive representation of the end-to-end supply chain network^42^. By integrating real-time data from multiple nodes and partners, VSCV enables firms to monitor, track, and assess operations at a granular level^43^. This transparency allows decision-makers to detect environmental inefficiencies, simulate green alternatives, and collaborate across functional and organizational boundaries^18^. With enhanced visibility, firms are better positioned to initiate and execute reconfiguration efforts such as modifying transportation routes, reengineering sourcing strategies, or adjusting production flows to improve environmental performance^44^. Thus, VSCV serves as a critical technological enabler of GPR. This relationship is underpinned by DCT which posits that the ability to sense environmental changes and reconfigure resources in response to them is essential for achieving sustainable innovation^16^.

H2a: Virtual supply chain visibility positively influences green process reconfiguration.

### Virtual supply chain visibility and eco-intelligent decision support

Effective sustainability in supply chains demands not only awareness of environmental challenges but also the ability to make decisions informed by accurate, real-time data across the supply network^1^. Yet many firms continue to make decisions with limited environmental intelligence due to fragmented systems, poor data integration, and lack of supply chain transparency^4^. As a result, decisions tend to prioritize cost or efficiency without accounting for environmental trade-offs^49^. This gap signals the need for EIDS, a capability that allows firms to systematically integrate sustainability considerations into their operational and strategic decision-making processes.

VSCV, as a core metaverse capability, enhances transparency by delivering an interactive, real-time view of supply chain activities, including those of suppliers, logistics providers, and downstream partners^42^. By consolidating data from disparate sources into a unified visual and analytical environment, VSCV enables firms to detect environmental risks, track sustainability metrics, and predict the outcomes of potential actions^43^. This real-time awareness supports more informed, sustainability-conscious decisions at all levels of the supply chain^18^. As such, VSCV directly contributes to the development of EIDS capabilities within organizations. This relationship aligns with DCT, which posits that organizations gain adaptive advantage by sensing environmental changes, integrating information, and reconfiguring decision processes to achieve sustainable outcomes^16^.

H2b: Virtual supply chain visibility positively influences eco-intelligent decision support.

### Green process reconfiguration and sustainable supply chain innovation

Innovation in sustainable supply chains requires firms to go beyond incremental improvements and actively redesign the core structures that drive operational impact^51^. While technologies and policies can guide sustainability transitions, true innovation arises when firms fundamentally reconfigure their processes, such as production, sourcing, and logistics to reduce environmental harm while maintaining or enhancing performance^27^. However, many firms lack the internal capabilities to initiate such a transformation^49^. This highlights the importance of GPR, which reflects a firm’s ability to strategically modify its supply chain architecture in line with sustainability goals.

By engaging in GPR, firms develop the capacity to evaluate, test, and implement greener alternatives to conventional operations. This includes rethinking workflows to reduce emissions, adopting circular material flows, and embedding eco-design principles into sourcing and manufacturing^25^. These structural changes allow firms to go beyond compliance and drive innovation that is both sustainability-oriented and performance-enhancing^52^. In doing so, GPR becomes a direct pathway through which internal transformation supports SSCI defined as the implementation of novel practices, processes, or strategies that advance environmental sustainability within the supply chain. This relationship is grounded in DCT, which posits that firms achieve sustainable innovation by continuously reconfiguring internal processes and resources to adapt to changing environmental and competitive conditions^25^.

H3: Green process reconfiguration positively influences sustainable supply chain innovation.

### Eco-intelligent decision support and sustainable supply chain innovation

Achieving innovation in sustainable supply chains increasingly depends on a firm’s ability to make decisions that are both strategically sound and environmentally informed^32^. Traditional decision-making models in supply chains often prioritize efficiency, speed, and cost while overlooking the ecological impact of operations^8^. As environmental regulations tighten and stakeholder expectations evolve, this outdated approach limits a firm’s capacity to innovate^49^. This underscores the growing importance of EIDS, which captures the ability to incorporate sustainability criteria into operational, tactical, and strategic decisions.

Firms equipped with EIDS are better positioned to assess the long-term environmental consequences of supply chain actions and to balance those considerations with operational and financial goals. Through predictive analytics, integrated data streams, and scenario evaluation, EIDS empowers managers to choose greener alternatives with greater confidence and strategic alignment^47^. As firms embed ecological intelligence into their decision-making infrastructure, they unlock new ways to develop and implement sustainable practices across the supply chain^48^. This transformation contributes directly to SSCI, enabling the organization to pursue novel, high-impact sustainability initiatives that are informed by real-time insights and holistic performance metrics. This relationship aligns with DCT, which asserts that organizations achieve innovation and long-term adaptability by continuously integrating, sensing, and reconfiguring decision-making processes in response to environmental changes^25^.

H4: Eco-intelligent decision support positively influences sustainable supply chain innovation.

### Digital maturity as a moderator between GPR and SSCI

While internal transformation mechanisms like GPR are essential for sustainability-driven innovation, their effectiveness is often contingent on an organization’s broader technological and cultural readiness. Even when firms possess the capability to reconfigure supply chain processes, the successful execution of these redesigns depends on having a digitally mature environment that supports experimentation, integration, and scalability^24^. Without sufficient infrastructure, digital skills, and strategic alignment, efforts to implement sustainability-oriented process changes may fail to produce meaningful or lasting innovation^21^.

DM reflects a firm’s readiness and capacity to deploy, manage, and scale digital technologies across its supply chain functions^23^. It encompasses not only technical assets but also organizational culture, cross-functional alignment, and leadership commitment to digital transformation^21^. In firms with higher DM, GPR is more likely to result in successful sustainable innovations, as these firms can leverage digital tools more effectively, align teams around green goals, and operationalize redesigned workflows with greater agility. In contrast, firms with low DM may face resistance, inefficiencies, or misalignment that reduce the impact of reconfiguration efforts on innovation outcomes. Grounded in DCT^16^, this relationship suggests that DM strengthens the transformation pathway by enhancing a firm’s ability to integrate digital and environmental objectives dynamically.

DM is conceptualized as a boundary condition that shapes how effectively GPR is translated into SSCI rather than how such reconfiguration is initiated. While metaverse-enabled capabilities activate internal transformation mechanisms, the successful implementation, scaling, and institutionalization of reconfigured processes depend on firms’ digital infrastructure, integration capacity, and strategic alignment, which are captured by digital maturity^21,53^. Accordingly, higher levels of DM strengthen the impact of GPR on innovation outcomes.

H5a: Digital maturity positively moderates the relationship between green process reconfiguration and sustainable supply chain innovation.

### Digital maturity as a moderator between EIDS and SSCI

The implementation of sustainability-oriented decision-making is not solely a function of available analytics or tools, it also depends heavily on the organization’s broader digital readiness and capability to act upon the intelligence generated^21^. Even when firms possess advanced data-driven decision support systems, their potential to enable sustainability innovations can be constrained by insufficient integration, fragmented systems, or lack of cross-functional cooperation^24^. These barriers limit the translation of eco-intelligent insights into strategic actions, weakening their impact on supply chain innovation^21^.

DM, in this context, plays a critical role by ensuring that firms have the technological infrastructure, strategic alignment, and cultural adaptability to fully utilize decision support systems^24^. In more digitally mature firms, decision-makers are better equipped to embed sustainability metrics into real-time decisions, coordinate actions across departments, and implement insights quickly and effectively^21^. This enhances the contribution of EIDS to SSCI, as sustainability becomes a strategic and integrated part of planning and execution. On the contrary, low DM may hinder responsiveness, delay execution, and reduce the impact of intelligent systems on innovative outcomes. Grounded in DCT^16^, this relationship suggests that digital maturity enhances a firm’s capacity to reconfigure its decision-making processes, enabling the dynamic alignment of environmental intelligence with innovation objectives^49^.

DM is conceptualized as a boundary condition that shapes how effectively EIDS is translated into SSCI rather than how such decision support is initially enabled. While EIDS can generate sustainability-oriented insights, their contribution to innovation outcomes depends on firms’ ability to integrate these insights into coordinated, real-time organizational actions, which are captured by digital maturity^16,21^.

H5b: Digital maturity positively moderates the relationship between eco-intelligent decision support and sustainable supply chain innovation.

### Control variables

To strengthen the reliability of the structural model and reduce potential bias, two control variables were included in this study: firm size and export orientation. These variables help account for organizational and contextual factors that may influence a firm’s ability to pursue SSCI, aside from the primary constructs under investigation.

Firm size reflects the level of resources, infrastructure, and operational capacity available within an organization. Larger firms generally have more financial strength, advanced systems, and human capital, which can support the adoption of digital tools and sustainability practices^25^. By considering firm size, the analysis distinguishes the impact of digital capabilities from advantages that stem from organizational scale.

Export orientation indicates whether a firm participates in international markets, where regulatory demands and customer expectations around sustainability are often more stringent. Firms engaged in exports are typically under greater pressure to adopt environmentally responsible practices and digital innovations to meet global standards^54^. Including export orientation as a control variable helps separate the effects of internal capabilities from external market-driven influences.

### Framework

**Fig. 1:**
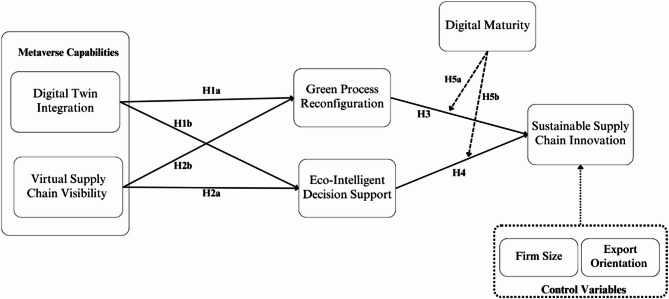
Conceptual framework.

The conceptual framework in Fig. 1, grounded in the DCT and TOE frameworks, illustrates how metaverse capabilities such as DTI and VSCV drive SSCI through two mediating mechanisms, GPR and EIDS. Digital maturity moderates the relationship between these mediators and SSCI, while firm size and export orientation are included as control variables to account for organizational scale and market context.

## Research methodology

### Research design

This study adopts a quantitative research design using a structured survey approach to examine the proposed conceptual model. The goal is to explore how metaverse capabilities influence SSCI through internal transformation mechanisms and moderated by organizational digital readiness. The collected data were analyzed using structural equation modeling (SEM) with WarpPLS 8.0, which is well-suited for assessing complex relationships among latent constructs and testing mediation and moderation effects^55^. WarpPLS was chosen because it supports both linear and nonlinear relationships among latent variables and is particularly appropriate for small- to medium-sized samples and non-normally distributed data. Moreover, it provides comprehensive model fit indices (APC, ARS, AVIF, AFVIF, GoF) that enhance the assessment of model quality. Additionally, to verify the stability of the results, a sensitivity check was conducted by randomly splitting the sample into two subsamples (n₁ = 215; n₂ = 215). The path coefficients remained consistent across both estimations, confirming the robustness of the findings.

#### Rationale for selecting Pakistan’s textile industry

The textile sector was purposefully selected as the empirical context for this study due to its economic importance and pressing sustainability challenges in Pakistan^26^. As one of the country’s largest contributors to exports, employment, and industrial output, the textile industry is highly resource-intensive and environmentally impactful^26^. Many firms in this sector struggle with outdated operational processes, low visibility across supply chain tiers, and limited use of advanced digital technologies^27,29^. Despite growing regulatory pressure and buyer expectations for greener practices, the sector often lacks the structural capability to implement sustainability-driven innovations^28,30^. These conditions align with our research model, which examines how metaverse capabilities enable SSCI through internal transformation, moderated by DM. Thus, Pakistan’s textile industry offers a timely and relevant context to explore digital transformation’s role in driving sustainability in under-digitized supply chains.

Similar studies in manufacturing-intensive industries such as automotive and electronics have shown that digitalization enhances transparency and resilience but often falls short in driving systemic sustainability innovation^56,57^. In contrast, most research within the textile sector remains focused on compliance and incremental process improvements, with limited exploration of advanced digital approaches that can simulate, visualize, and reconfigure supply chain processes in real time capabilities that metaverse technologies uniquely provide for driving proactive and system-wide sustainability transformation^58–60^. The present study therefore extends this body of work by demonstrating how advanced digital capabilities can enable proactive, sustainability-driven innovation in under-digitized industrial contexts.

### Instrument development

All items were measured using a five-point Likert scale ranging from 1 (strongly disagree) to 5 (strongly agree). The measurement items for all constructs were derived following the C-OAR-SE framework^61^. A comprehensive review of prior studies informed item generation to ensure content validity, conceptual distinctiveness, and coverage of the construct domains. Item wording was refined for clarity and contextual relevance, and peer feedback and expert review were incorporated to confirm face validity. Aligning with this methodology, items for DTI were developed from research on digital manufacturing and twin capabilities^17,45^, while VSCV was created by immersive analytics and supply chain transparency study^18^. The constructs for GPR and EIDS were newly developed in this study but grounded in process innovation and sustainability analytics literature^25,46–48,52^. DM was assessed using items that reflect organizational infrastructure, system integration, and leadership alignment, based on digital readiness and transformation frameworks^23,24,53^. SSCI was measured using innovation indicators tied to environmental goals and sustainable operations^51^. A pilot test was conducted with 30 supply chain professionals to refine the instrument in terms of clarity and contextual relevance. Based on their feedback, minor revisions were made.

### Data collection

Prior to the main survey, the questionnaire was pretested to ensure clarity, content validity, and contextual relevance. The initial instrument was reviewed by academic experts and industry professionals with experience in supply chain management and digital transformation, leading to minor refinements in wording and item sequencing. A pilot test was subsequently conducted with a small group of respondents to assess item comprehensibility and completion time. To reduce potential common method bias, procedural remedies were applied, including assuring respondent anonymity, minimizing evaluation apprehension, and separating measurement of predictor and criterion variables in the questionnaire design.

The primary data were collected from firms within the textile manufacturing and exporting sector in Pakistan, a critical and sustainability-sensitive industry. Data were gathered from the top three textile-producing cities in Pakistan, namely Karachi, Faisalabad, and Lahore, to ensure a broad and regionally representative sample^62^. The survey targeted professionals in roles such as supply chain managers, digital transformation leads, operations directors, and sustainability officers. A stratified purposive sampling technique was employed to capture variation across firms with different levels of DM. Both online and in-person distribution methods were utilized to administer the questionnaire. Data collection was carried out between April 2025 to May 2025. After screening for completeness and consistency, a total of 430 valid responses were retained for final analysis.

The demographic characteristics of the respondents are summarized in Table 1. The sample consisted of 320 males (74.42%) and 110 females (25.58%). In terms of age, 120 participants (27.91%) were aged 20–29, 180 (41.86%) were aged 30–39, and 130 (30.23%) were aged 40 years and above. In terms of firm size, 60 respondents (13.95%) were from small firms (1–49 employees), 140 (32.56%) from medium-sized firms (50–249 employees), and 230 (53.49%) from large firms (250 or more employees). Regarding job positions, 160 respondents (37.21%) were managers, 190 (44.19%) were executives, and 80 (18.60%) were directors. For professional experience, 100 respondents (23.26%) had less than 5 years of experience, 180 (41.86%) had between 5 and 10 years, and 150 (34.88%) had more than 10 years of experience. Regarding export orientation, 290 respondents (67.44%) were from export-oriented firms, while 140 (32.56%) represented non-exporting firms. These distributions reflect a diverse and well-balanced sample suitable for investigating DM and sustainability-driven innovation in Pakistan’s textile sector.

**Table 1:** Demographic characteristics.

Category	Subcategory	Frequency (*n*)	Percentage (%)
Gender	Male	320	74.42%
	Female	110	25.58%
Firm age	20–29 years	120	27.91%
	30–39 years	180	41.86%
	40 years and above	130	30.23%
Firm size	Small (1–49 employees)	60	13.95%
	Medium (50–249)	140	32.56%
	Large (250 + employees)	230	53.49%
Job position	Manager	160	37.21%
	Executive	190	44.19%
	Director	80	18.60%
Work experience	Less than 5 years	100	23.26%
	5–10 years	180	41.86%
Export orientation	Exporting firm	290	67.44%
	Non-exporting firm	140	32.56%

## Data evaluation and findings

### Common method bias (CMB)

To assess the potential for common method bias, Harman’s single-factor test was conducted. The unrotated principal component analysis showed that the first factor accounted for only 34.7% of the total variance, well below the 50% threshold, suggesting that common method bias was not a significant concern in this study. Additionally, a full collinearity variance inflation factor (VIF) test was conducted, with all VIF values below the critical value of 3.3, further indicating the absence of CMB issues^63^.

### Model fit and quality indices

The model was assessed using WarpPLS 8.0, demonstrating strong overall fit, summersized in Table 2. The Average Path Coefficient (APC) was 0.294 (*p* < 0.001), and the Average R-squared (ARS) was 0.367 (*p* < 0.001), indicating a well-fitting model. Adjusted R-squared (AARS) was 0.353 (*p* < 0.001). Average block VIF (AVIF = 1.72) and Average full collinearity VIF (AFVIF = 2.14) were within acceptable thresholds (≤ 5). Tenenhaus GoF was 0.496, reflecting a large effect size. Other quality indices including SPR (1.00), RSCR (1.00), SSR (1.00), and NLBCDR (0.96) indicated strong predictive relevance.

**Table 2:** Model fit and quality indices.

Fit indices	Value	Threshold	Interpretation
Average path coefficient (APC)	0.294	*p* < 0.001	Significant
Average R-squared (ARS)	0.367	*p* < 0.001	Moderate explanatory power
Adjusted average R-squared (AARS)	0.353	*p* < 0.001	Moderate explanatory power
Average block VIF (AVIF)	1.72	≤ 5.00	Acceptable collinearity
Average full collinearity VIF (AFVIF)	2.14	≤ 5.00	Acceptable collinearity
Tenenhaus GoF	0.496	≥ 0.36 (large)	Large effect size
Sympson’s Paradox Ratio (SPR)	1.00	> 0.70	Ideal
R-squared Contribution Ratio (RSCR)	1.00	> 0.90	Excellent contribution
Statistical Suppression Ratio (SSR)	1.00	> 0.70	Ideal
Nonlinear Bivariate Causality Direction Ratio (NLBCDR)	0.96	> 0.70	Strong predictive relevance

### Measurement model assessment

Convergent validity was confirmed as all item loadings exceeded the recommended threshold of 0.70, indicating that each item adequately reflects its associated construct. Average variance extracted (AVE) values for all constructs were above 0.50, further supporting convergent validity. Composite reliability (CR) values ranged from 0.88 to 0.93, demonstrating internal consistency and reliability of the measurement scales.

Particular emphasis was placed on validating the newly developed constructs GPR and EIDS. Both constructs exhibited strong psychometric properties, with all factor loadings above 0.80, AVE values exceeding 0.70, and CR values above 0.90. These results confirm their convergent validity and reliability. In addition, discriminant validity tests (Fornell Larcker and HTMT) confirmed that GPR and EIDS are empirically distinct yet conceptually related, validating them as independent constructs representing process and decision level transformation mechanisms.

Discriminant validity was assessed using both the fornell-larcker criterion and heterotrait-monotrait (HTMT) ratio. According to the Fornell-Larcker approach, the square root of AVE for each construct was greater than its correlations with other constructs, establishing discriminant validity. Additionally, all HTMT values were below the conservative threshold of 0.85, reinforcing the distinctiveness of each construct. Furthermore, multicollinearity diagnostics indicated acceptable levels, as all item-level variance inflation factor (VIF) values were below the threshold of 3.3. This suggests that multicollinearity was not a concern in the measurement model. The detailed results of the measurement model including factor loadings, AVE, CR, and VIF values and discriminant validity are presented in Tables 3 and 4.

**Table 3:** Measurement model assessment.

Construct	Item code	Loading	AVE	CR	VIF
DTI	DTI1	0.84	0.74	0.89	2.14
	DTI2	0.87			2.29
	DTI3	0.88			2.23
VSCV	VSCV1	0.85	0.76	0.90	2.17
	VSCV2	0.89			2.36
	VSCV3	0.87			2.28
GPR	GPR1	0.83	0.71	0.90	2.06
	GPR2	0.86			2.14
	GPR3	0.84			2.09
	GPR4	0.85			2.11
EIDS	EIDS1	0.88	0.73	0.91	2.32
	EIDS2	0.84			2.26
	EIDS3	0.85			2.20
	EIDS4	0.87			2.24
DM	DM1	0.83	0.72	0.88	2.12
	DM2	0.87			2.18
	DM3	0.86			2.14
SSCI	SSCI1	0.86	0.74	0.93	2.38
	SSCI2	0.87			2.42
	SSCI3	0.88			2.35
	SSCI4	0.85			2.40
	SSCI5	0.89			2.45

Digital twin integration (DTI), Virtual supply chain visibility (VSCV), Green process reconfiguration (GPR), Eco-intelligent decision support (EIDS), Digital maturity (DM), Sustainable supply chain innovation (SSCI).

**Table 4:** Discriminant validity: fornell-larcker criterion.

Construct	DTI	VSCV	GPR	EIDS	DM	SSCI
DTI	0.82					
VSCV	0.61	0.81				
GPR	0.52	0.54	0.83			
EIDS	0.48	0.51	0.59	0.84		
DM	0.43	0.45	0.53	0.58	0.81	
SSCI	0.55	0.56	0.62	0.61	0.59	0.84

### Structural model and hypothesis testing

All hypothesized relationships were statistically supported, as presented in Table 5. DTI significantly enhanced GPR (β = 0.416, T = 6.72, *p* < 0.001) and EIDS (β = 0.384, T = 5.38, *p* < 0.001). Similarly, VSCV positively influenced GPR (β = 0.355, T = 4.91, *p* < 0.001) and EIDS (β = 0.398, T = 7.02, *p* < 0.001). Both GPR (β = 0.364, T = 4.23, *p* < 0.001) and EIDS (β = 0.421, T = 5.97, *p* < 0.001) significantly contributed to sustainable supply chain innovation. Moreover, DM positively moderated the effects of GPR (β = 0.137, T = 2.17, *p* = 0.029) and EIDS (β = 0.159, T = 2.74, *p* = 0.016) on SSCI, underscoring the importance of digital readiness in realizing sustainability outcomes.

**Table 5:** Structural model results and hypothesis testing.

Hypothesis	Path	β	T-value	*p*-value	Result
H1a	DTI → GPR	0.416	6.72	< 0.001	Supported
H1b	DTI → EIDS	0.384	5.38	< 0.001	Supported
H2a	VSCV → GPR	0.355	4.91	< 0.001	Supported
H2b	VSCV → EIDS	0.398	7.02	< 0.001	Supported
H3	GPR → SSCI	0.364	4.23	< 0.001	Supported
H4	EIDS → SSCI	0.421	5.97	< 0.001	Supported
H5a	GPR × DM → SSCI	0.137	2.17	0.029	Supported
H5b	EIDS × DM → SSCI	0.159	2.74	0.016	Supported

### Indirect effects

All hypothesized indirect effects were statistically supported, as shown in Table 6. H6a confirmed that GPR significantly mediated the relationship between DTI and SSCI (β = 0.151, T = 3.92, *p* < 0.001), while H6b supported its mediating role between VSCV and SSCI (β = 0.129, T = 3.48, *p* < 0.001). Similarly, H6c validated that EIDS mediated the effect of DTI on SSCI (β = 0.162, T = 4.15, *p* < 0.001), and H6d confirmed its mediating role between VSCV and SSCI (β = 0.167, T = 4.31, *p* < 0.001). These results highlight the centrality of internal transformation mechanisms in translating metaverse capabilities into SSCI.

**Table 6:** Mediation analysis.

Hypothesis	Indirect Path	β	T-value	*p*-value
H6a	DTI → GPR → SSCI	0.151	3.92	< 0.001
H6b	VSCV → GPR → SSCI	0.129	3.48	< 0.001
H6c	DTI → EIDS → SSCI	0.162	4.15	< 0.001
H6d	VSCV → EIDS → SSCI	0.167	4.31	< 0.001

## Discussion

The empirical results provide strong support for our conceptual model, demonstrating that metaverse capabilities play a pivotal role in driving SSCI. Specifically, both DTI and VSCV exhibited significant positive effects on the internal transformation mechanisms GPR and EIDS. These findings align with and extend previous research that highlighted the limitations of traditional digital tools like ERP and IoT, which often lack the real-time simulation and immersive visibility required for sustainability-oriented redesign^5,8,9,40^. By introducing more advanced digital functionalities through the metaverse, our study confirms that firms can move beyond static process improvements to actively redesign operations in support of environmental goals. This helps textile manufacturers in Pakistan address their long-standing inefficiencies and lack of visibility across supply chain tiers, enabling more proactive and strategic sustainability management.

Moreover, the mediating roles of GPR and EIDS were statistically significant, suggesting that the influence of metaverse capabilities on SSCI is not direct but operates through the transformation of internal capabilities. These mediators provided the organizational pathway through which immersive technologies translate into tangible innovation outcomes. Notably, these mediation effects represent a novel contribution, as prior studies have largely examined digital transformation in isolation without unpacking the specific process mechanisms involved^18,20,64^. Our findings suggest that metaverse-driven technologies foster experimentation, environmental intelligence, and proactive reconfiguration, offering more dynamic and scalable benefits than conventional digital systems studied in earlier work. This enables Pakistan’s textile sector to move beyond compliance-based approaches and achieve deeper environmental innovation within core supply chain processes.

Finally, the moderating role of DM adds a critical boundary condition to these relationships. The interaction effects revealed that the benefits of both GPR and EIDS on SSCI are significantly stronger in firms with higher levels of digital readiness. This result highlights the importance of organizational infrastructure, leadership commitment, and digital culture in amplifying the effectiveness of metaverse capabilities. Compared to baseline studies that treat DM as a background characteristic, our findings underscore its strategic role in enhancing sustainability outcomes^21,24^. These insights not only validate the relevance of the DCT in this context but also position DM as a crucial lever for firms seeking to maximize the return on their metaverse investments in pursuit of sustainable innovation. This insight is particularly valuable for Pakistan’s textile firms, many of which operate with uneven digital capacity, helping them understand how readiness can enhance technology-driven sustainability transitions.

In addition, firm size and export orientation were included as control variables to account for organizational and contextual variability. The results revealed that firm size had a significant positive effect on SSCI, suggesting that larger firms, with greater access to resources and infrastructure, are more capable of adopting advanced metaverse technologies and implementing sustainability-focused innovations. Similarly, export orientation also showed a positive and significant influence on SSCI, indicating that firms engaged in international markets are more likely to pursue sustainability initiatives, possibly due to stricter regulatory compliance and competitive pressures in global supply chains. These findings underscore the relevance of external structural factors in shaping the effectiveness of digital transformation efforts and highlight the need for tailored strategies when promoting SSCI across firms of different sizes and market scopes.

### Theoretical contributions

This study offers a substantial theoretical contribution by enriching the TOE framework^15^, which posits that the adoption and impact of technological innovations are shaped by the interplay of technological, organizational, and environmental contexts. From the technological dimension, our research emphasizes advanced metaverse capabilities DTI and VSCV as critical enablers that go beyond conventional digital tools by offering immersive, real-time modeling and visibility functions. In the organizational dimension, we highlight the role of DM, which encompasses a firm’s infrastructure readiness, leadership support, and ability to integrate digital technologies into daily operations. This moderating role underscores how internal readiness conditions the effectiveness of digital transformation in driving sustainable innovation. Finally, the environmental dimension is captured through the industry’s external pressures, such as climate regulations, buyer expectations, and sustainability demands, particularly in the context of Pakistan’s textile sector. By integrating all three dimensions, our study extends TOE theory by demonstrating how these contextual elements collectively shape the pathway from digital capability to sustainability-driven supply chain innovation. This contribution moves TOE-based digital transformation research beyond technology adoption toward explaining how contextual alignment enables sustainability-oriented outcomes.

In addition to the TOE framework, this study also builds on DCT^16^, by conceptualizing and empirically validating DM as a key moderator that strengthens a firm’s ability to leverage digital technologies for sustainable innovation. DCT suggests that organizations must continually reconfigure internal competencies and resources to respond to rapidly changing environments. In our study, DM reflects this adaptive capacity, embodying a firm’s ability to sense technological opportunities, seize them through aligned leadership and infrastructure, and reconfigure processes to support transformation. The moderating effect of DM on the relationship between both GPR and EIDS with SSCI confirms that such transformation is not solely technology-driven but also contingent on a firm’s internal absorptive capacity. By embedding DM within a dynamic capabilities lens, our findings advance DCT by showing how this capability enables firms to convert metaverse-enabled insights into meaningful, sustainability-focused process innovations. Together with TOE, this dynamic capabilities perspective clarifies how metaverse-enabled technologies are translated into operational and decision-level transformation rather than remaining isolated digital tools.

### Practical implications

This study offers several actionable insights for practitioners aiming to advance sustainability in complex and under-digitized supply chains, particularly within emerging economy contexts like Pakistan’s textile industry. First, managers should prioritize the deployment of metaverse-driven technologies such as DTI and. These capabilities enable immersive simulations, real-time modeling, and system-wide visibility, empowering firms to experiment with greener strategies without disrupting operations. Firms can use these technologies to identify inefficiencies, test eco-friendly process alternatives, and improve cross-tier coordination all of which are essential for achieving SSCI.

Second, the findings emphasize that simply adopting advanced technologies is not sufficient. Organizations must also invest in internal capabilities that support structural transformation and intelligent planning. The two identified mediating mechanisms GPR and EIDS highlight the importance of redesigning processes and integrating environmental data into decision-making routines. Firms should build cross-functional teams capable of environmental experimentation, embed sustainability metrics into their digital dashboards, and adopt agile workflows that support continuous improvement in environmental performance.

Finally, DM emerged as a critical enabler of successful transformation. Firms with higher levels of DM were more effective in translating digital insights into sustainable outcomes. This suggests that firms should not only focus on technology acquisition but also strengthen their digital infrastructure, leadership alignment, and innovation culture. Training programs, change management initiatives, and integrated digital roadmaps can all help organizations scale sustainability-focused digital transformations. For the textile sector in particular, where resource constraints and environmental scrutiny are high, these efforts can significantly improve operational resilience, reduce regulatory risk, and enhance competitive positioning in sustainability-conscious global markets. Beyond Pakistan’s textile industry, these practical insights are relevant to other manufacturing and logistics-intensive sectors, such as automotive, electronics, and energy, where digital transformation and sustainability goals increasingly converge. The mechanisms identified DTI, VSCV, GPR, and EIDS offer transferable strategies for organizations in both emerging and developed economies to enhance sustainable supply chain innovation and environmental performance.

### Policy implications

The findings of this study carry meaningful implications for public policymakers, industry regulators, and sustainability-focused institutions working to accelerate the green transition in Pakistan’s textile sector a major contributor to national GDP and environmental impact.

First, the government and industry bodies should incentivize digital sustainability investments by offering tax credits, grants, or low-interest financing programs for firms adopting advanced technologies such as DTI and VSCV. These tools have demonstrated potential to promote simulation-based process redesign and real-time environmental intelligence, both of which are crucial for green innovation. Second, DM emerges as a critical enabler of sustainability outcomes. Policymakers should therefore develop sector-specific digital transformation roadmaps and support programs that enhance digital infrastructure, workforce digital literacy, and technology integration capabilities in small and medium enterprises. This includes facilitating public-private partnerships to deliver targeted digital upskilling and supply chain innovation training. Third, to encourage adoption of eco-intelligent practices, regulators can mandate sustainability disclosure frameworks that require firms to embed environmental metrics into operational decisions. This would create demand for EIDS systems and accelerate internal sustainability transformation. Finally, the national export and trade policy can be strategically aligned to reward SSCI. By linking export incentives or preferential treatment to digital sustainability adoption, Pakistan can enhance the global competitiveness of its textile industry while meeting international buyer and environmental standards.

In summary, supportive policies that promote technological innovation, organizational capability building, and sustainability enforcement can collectively create an ecosystem where firms are empowered and incentivized to pursue SSCI at scale. While these policy recommendations are derived from the textile sector, they hold broader relevance for policymakers in other industries and regions aiming to accelerate sustainable digital transformation. Similar policy instruments such as fiscal incentives for metaverse-driven technologies, national digital upskilling programs, and sustainability-linked trade policies can be adapted across diverse industrial contexts to strengthen green innovation capacity globally.

## Conclusion

This study investigated how metaverse-enabled digital capabilities specifically DTI and VSCV facilitate SSCI by activating internal transformation mechanisms, namely GPR and EIDS. Drawing on the DCT and the TOE framework, the research provides a comprehensive understanding of the pathways through which emerging digital technologies can contribute to sustainability-oriented innovations in complex and under-digitized sectors.

Empirical results from 430 textile firms in Pakistan confirm that both DTI and VSCV significantly influence SSCI, and that their effects are mediated by GPR and EIDS. The findings also highlight that DM plays a positive moderating role, enhancing the impact of internal transformation mechanisms on innovation outcomes. This reinforces the notion that the success of advanced digital capabilities depends not only on their adoption but also on the organization’s readiness to absorb and operationalize them strategically.

By focusing on Pakistan’s textile industry, a sector marked by high environmental exposure, fragmented digital infrastructure, and growing regulatory pressures, this study contributes novel theoretical and practical insights. The findings suggest that immersive and real-time technologies hold considerable potential to reduce the structural rigidity of traditional systems, helping firms explore process redesign, integrate ecological intelligence, and support innovation aligned with sustainability goals. The study offers a framework that may inform similar analyses in other emerging economies and sustainability-critical industries. Additionally, the significant influence of firm size and export orientation underscores the importance of organizational scale and global market engagement in shaping sustainability-driven digital transformation.

### Limitations and future research directions

While this study offers valuable insights into how metaverse-enabled capabilities drive sustainable supply chain innovation, several limitations should be acknowledged. First, the cross-sectional survey design captures relationships at a single point in time, limiting causal inference. Future studies could employ longitudinal or experimental approaches to examine how these relationships evolve as digital capabilities and sustainability practices mature. Second, the focus on Pakistan’s textile industry, while theoretically relevant for studying sustainability under resource constraints, may limit external validity due to its specific institutional, technological, and policy conditions. Accordingly, the study does not aim for statistical generalization across sectors or economies. Instead, it offers analytically transferable insights into how metaverse-enabled capabilities activate internal transformation mechanisms and how digital maturity conditions their effectiveness. Future research should validate the proposed framework across different industries and economic contexts to assess contextual transferability.

Third, macro-level challenges such as economic volatility, energy constraints, and political instability may have influenced firms’ digital and sustainability decisions. Incorporating macroeconomic or policy variables in future studies could help capture these external contingencies. Finally, the adoption of metaverse technologies may be constrained by technological, organizational, and regulatory barriers, including interoperability issues, skills shortages, and compliance requirements. Future research should explore how firms and policymakers can address these challenges to support effective implementation.

## Supplementary Information

Below is the link to the electronic supplementary material.

Supplementary Material 1

## Data Availability

The data supporting the findings of this study are available from the corresponding author upon reasonable request.
