# Supplementary material for: How metaverse-enabled digital transformation drives sustainable supply chain innovation: evidence from Pakistan’s textile industry
Source: Sci Rep. 2026 Mar 11;16:9098. doi: 10.1038/s41598-026-40819-6 (PMC12996279; doi:10.1038/s41598-026-40819-6)
Supplement: Supplementary file 1 — Supplementary Material 1 [file 41598_2026_40819_MOESM1_ESM.docx]

**Measurement Items for Research Constructs**

| **Construct** | **Item Code** | **Measurement Item** |
| --- | --- | --- |
| **Digital twin integration** ^17,45^ | DTI1 | Our operations benefit from having models that reflect real-world supply chain activity. |
|  | DTI2 | We explore different scenarios to improve supply chain outcomes before making changes. |
|  | DTI3 | Real-time digital tools support how we monitor and improve operational performance. |
| **Virtual supply chain visibility** ^18^ | VSCV1 | Our teams can access supply chain activities across all levels through digital systems. |
|  | VSCV2 | Data from various partners is available in a format that supports informed decisions. |
|  | VSCV3 | Real-time visibility helps us understand environmental impact across the supply chain. |
| **Green process reconfiguration** ^25,46,52^ | GPR1 | Our processes are regularly updated to align with environmental and efficiency goals. |
|  | GPR2 | Teams test new alternatives in logistics and production for better sustainability. |
|  | GPR3 | Operations are flexible to accommodate evolving sustainability requirements. |
|  | GPR4 | Environmental goals are considered when rethinking supply chain processes. |
| **Eco-intelligent decision support** ^46–48^ | EIDS1 | Environmental factors are integrated into routine supply chain decisions. |
|  | EIDS2 | Predictive tools help identify and plan for environmental risks. |
|  | EIDS3 | Decisions reflect a balance between ecological and operational priorities. |
|  | EIDS4 | Data-driven systems are used to support sustainability-focused planning. |
| **Digital maturity** ^23,24,53^ | DM1 | Our digital systems support coordination across supply chain functions. |
|  | DM2 | Leadership encourages the use of digital tools for sustainability goals. |
|  | DM3 | Infrastructure supports scaling of new digital technologies organization-wide. |
| **Sustainable supply chain innovation** ^51^ | SSCI1 | New practices are introduced to align supply chain operations with sustainability goals. |
|  | SSCI2 | Environmental aspects are included when planning supply chain innovations. |
|  | SSCI3 | Our supply chain innovations aim to improve both performance and sustainability. |
|  | SSCI4 | We explore ideas that lead to more sustainable products or processes. |
|  | SSCI5 | Our teams contribute to advancing sustainability within the supply chain field. |
